# Anaphylaxis with delayed appearance of skin manifestations during general anesthesia: two case reports

**DOI:** 10.1186/s13104-017-2624-7

**Published:** 2017-07-24

**Authors:** Hiroshi Hanamoto, Fumi Kozu, Aiko Oyamaguchi, Mika Inoue, Chizuko Yokoe, Hitoshi Niwa

**Affiliations:** 1 Department of Dental Anesthesiology, Osaka University Graduate School of Dentistry, 1-8, Yamadaoka, Suita, Osaka 565-0871 Japan; 2 Department of Anesthesiology, Graduate School of Dentistry, Kanagawa Dental University, Yokosuka, Japan

**Keywords:** Anaphylaxis, General anesthesia, Skin manifestations, Respiratory symptoms

## Abstract

**Background:**

Anaphylaxis is difficult to diagnose in the absence of skin or mucosal signs and symptoms. We report two cases of anaphylaxis under general anesthesia, in which the initial presentation was in the form of respiratory signs, followed by skin manifestations 10–15 min later. Diagnosis of anaphylaxis was delayed because skin symptoms were absent early on in the presentation.

**Case presentation:**

In the first case, a 23-year-old male patient with jaw deformity was scheduled to undergo maxillary alveolar osteotomy. After intubation, auscultation indicated a sudden decrease in breath sounds, together with severe hypotension. Approximately 10 min later, flushing of the skin and urticaria on the thigh appeared and spread widely throughout the body. In the second case, a 21-year-old female patient with jaw deformity was scheduled to undergo maxillomandibular osteotomy. Twenty minutes after the start of dextran infusion, her lungs suddenly became difficult to ventilate, and oxygen saturation decreased to 90%. Approximately 15 min later, flushing of the skin and urticaria were observed.

**Conclusion:**

In both cases, there was a time lag between the appearance of respiratory and skin symptoms, which resulted in a delay in the diagnosis, and hence, treatment of anaphylaxis. Our experience highlights the fact that it is difficult to diagnose anaphylaxis under general anesthesia.

## Background

Generally, anaphylaxis is diagnosed on the basis of cutaneous, respiratory, circulatory, and gastrointestinal symptoms and signs [1]. Cutaneous manifestations, such as urticaria and angioedema, are the most common symptoms of anaphylaxis [2–5] and often present in the initial stages of severe anaphylaxis [2]. Skin manifestations are key to making a diagnosis of anaphylaxis. Since 80–90% of patients develop cutaneous manifestations, it is difficult to diagnose anaphylaxis without them [1].

Anaphylaxis is one of the most severe adverse events during general anesthesia. It is more difficult to diagnose anaphylaxis in anesthetized patients than in awake patients, because there are no complaints from the patient, and many other disorders with similar symptoms and signs may occur under general anesthesia, confounding the diagnosis [6]. We report two cases of anaphylaxis under general anesthesia with initial respiratory symptoms and delayed skin manifestations.

## Case presentation

### Case 1

A 23-year-old, 169-cm, 53-kg male patient with jaw deformity was scheduled to undergo maxillary alveolar osteotomy. He had no history of allergy to any drugs or foods. Preoperative examination, including laboratory data, chest X-ray and electrocardiogram (ECG) were normal. No premedication was administered. After arriving at the operation room, arterial blood pressure (BP), heart rate (HR) and oxygen saturation (SpO_2_) were 152/85 mmHg, 71 bpm and 100%, respectively.

After venous access was obtained, 2 mg of midazolam (Dormicum; Astellas, Tokyo, Japan) and 50 μg of fentanyl (Daiichi Sankyo, Tokyo, Japan) were administered. Then, a nasogastric tube was inserted under sedation. At this time, the patient’s BP, HR and SpO_2_ were 147/82 mmHg, 67 bpm and 100%, respectively. Anesthesia was induced with a target controlled infusion (TCI) of 3 μg/ml of propofol (Diprivan; AstraZeneca K.K., Osaka, Japan) and 0.25 μg/kg/min of remifentanil (Ultiva; Janssen Pharmaceutical K.K., Tokyo, Japan). After mask ventilation with 100% oxygen, 40 mg of rocuronium (Eslax; MSD K.K., Tokyo, Japan) was administered to facilitate tracheal intubation. Nasotracheal intubation was performed uneventfully and confirmed by breath sound auscultation and capnography. Just after intubation, the remifentanil dose was decreased to 0.1 μg/kg/min.

A few minutes later, the anesthetist who was continuously listening to the respiratory sounds with a stethoscope suddenly noticed diminution of the respiratory sounds all over the patient’s chest 5 min after the administration of rocuronium. At this time, the patient’s lungs were manually ventilated. Although we do not have accurate data of peak inspiratory pressure (PIP) at that time, movement of the thoracic cage is limited with a PIP of ≥15–20 cm H_2_O. SpO_2_ was 99–100% throughout the event. Although we did consider the possibility of asthma, a beta-2 stimulant was not administered because wheezing was not identified. Although the trachea was suctioned and lungs were inflated again, the respiratory sounds did not improve. Four minutes after appearance of the respiratory symptoms, his BP dropped to 43/21 mmHg and HR was 89 bpm. Administration of 4 mg of ephedrine intravenously together with fluid therapy had no effect on the hypotension. As his BP and HR remained at 41/24 mmHg and 88 bpm respectively, administration of propofol and remifentanil were discontinued and an additional 4 mg of ephedrine was administered. Although the patient’s BP transiently increased to 69/33 mmHg (HR was 94 bpm), there was no further effect. Then, flushing of the skin over the chest and urticaria on the thigh emerged and spread widely all over the body over a few minutes. The time interval between emergence of the initial respiratory symptoms and appearance of skin manifestations was approximately 10 min.

According to the recommended treatment for anaphylaxis, 100% oxygen was supplied and 50 µg of adrenaline (Bosmin; Daiichi Sankyo), 5 mg of chlorpheniramine (Polaramine; MSD K.K.) and 500 mg of methylprednisolone (Solu-Medrol; Pfizer Japan, Tokyo, Japan) were administered. With this treatment, the patient’s BP and respiratory sounds gradually improved over several minutes and the systemic flush and urticaria disappeared approximately 30 min after their appearance.

Since rocuronium was strongly suspected as the allergen from the time course of events, remifentanil was carefully re-administered and sevoflurane was administered. Administration of rocuronium and other muscle relaxants was discontinued. Surgery was performed without any complications. Serum tryptase levels, which were measured approximately 30 min and 24 h after the administration of rocuronium, were 12.0 and 1.1 μg/l, respectively.

### Case 2

A 21-year-old, 158-cm, 49-kg female patient with jaw deformity was scheduled to undergo maxillomandibular osteotomy. She had previously undergone palatoplasty under general anesthesia without any complications when she was 20 months old. She had no allergic history. Preoperative examination, including laboratory data, chest X-ray and ECG, was normal.

After sedation with 2 mg of midazolam and 50 μg of fentanyl, anesthesia was induced with 3 μg/ml of propofol (TCI) and 0.25 μg/kg/min of remifentanil. Nasotracheal intubation was successfully performed 2 min after 35 mg of rocuronium was administered. Anesthesia was maintained with 2–4 μg/ml of propofol and 0.1–0.3 μg/kg/min of remifentanil. For deliberate hypotension, 0.15 μg/kg/min of sodium nitroprusside (Nitopro; Maruishi Pharmaceutical Co. Ltd., Osaka, Japan) was continuously administered and BP was maintained at approximately 80/40 mmHg. Four hours after commencement of the surgery, the blood loss volume was approximately 350 ml, with more blood loss being anticipated. The patient’s BP and HR was 79/39 mmHg and 81 bpm, respectively. Hence, dextran 40 (Low Molecular Dextran L; Otsuka, Tokushima, Japan) was infused for replacement of the blood lost. Approximately 20 min after the start of dextran 40 infusion, the patient’s lungs suddenly became difficult to ventilate, and SpO_2_ decreased to 90%. After 100% oxygen was supplied and her lungs were adequately ventilated, SpO_2_ increased transiently to 98%. We asked the surgeons to stop the procedure and pulled off the drapes covering the patient. Lung sounds were difficult to hear on auscultation. However, evaluation revealed no problem with the endotracheal tube, and there were no skin manifestations that suggested anaphylaxis. Four minutes after appearance of the respiratory symptoms, the patient’s BP decreased to 60/20 mmHg and HR was 88 bpm. Hence, nitroprusside was discontinued and 0.2 mg of phenylephrine (Neo-Synesin; Kowa Pharmaceutical Co. Ltd., Nagoya, Japan) was administered. Fluid therapy was also administered. With these measures, although her BP transiently increased to 80/47 mmHg (HR was 87 bpm), it decreased once again to 61/42 mmHg (HR was 87 bpm) 10 min after the appearance of respiratory symptoms. Therefore, three additional 0.2 mg doses of phenylephrine were administered, and propofol and remifentanil were discontinued. Despite this, however, the hypotension did not improve. During the treatment of hypotension, SpO_2_ gradually decreased to 85%. Considering the possibility of tension pneumothorax, we commenced preparations for taking a chest X-ray. However, before we could do so, flushing of the skin and urticaria appeared on her thigh and spread widely over her entire body. The time interval from appearance of the first respiratory symptoms to that of skin manifestations was approximately 15 min.

According to the recommended treatment for anaphylaxis, 0.5 mg of adrenaline was administered intramuscularly, and 5 mg of chlorpheniramine and 500 mg of methylprednisolone were administered intravenously. Thereafter, ventilation and BP gradually improved over several minutes, and the systemic flush and urticaria disappeared approximately 30 min after their emergence. Anesthetics were re-administered and surgery was recommenced without any complications. In this patient, we considered dextran to be the offending allergen because of the time course of anaphylactic symptoms in relation to dextran administration. Postoperatively, the patient was transferred to the recovery room and sedated with midazolam and dexmedetomidine. She was successfully extubated the following day without any complications. Serum tryptase levels, which were measured approximately 30 min and 24 h after the administration of dextran 40, were 6.5 and 1.4 μg/l, respectively.

## Discussion

In both the above cases, the initial clinical manifestation was in the form of respiratory symptoms. Since skin manifestations developed 10–15 min after the respiratory and cardiovascular symptoms, the diagnosis of anaphylaxis was uncertain and delayed.

The incidence of anaphylaxis during general anesthesia reportedly ranges from 1 in 4000 to 1 in 25,000 [7], while the mortality from anesthesia-related anaphylaxis is estimated to be as high as 6% [2]. Although anaphylaxis during general anesthesia is rare, its early diagnosis and treatment are essential since anaphylaxis may progress rapidly to a life-threatening condition.

Diagnosis of anaphylaxis under general anesthesia is difficult for the following reasons. First, patients cannot complain about their symptoms, such as malaise, pruritus, dizziness and dyspnea. Second, cutaneous symptoms are often difficult to detect because they may be masked by the surgical drapes. Third, several other differential diagnoses for the symptoms, such as problems with the airway, cardiogenic and hemorrhagic shock, and pneumothorax during general anesthesia, have to be considered and ruled out. Although skin and mucosal symptoms and signs are key to the diagnosis of anaphylaxis, the incidence of their occurrence is lower among anesthetized patients than among awake patients [8]. Therefore, it is very difficult to diagnose anaphylaxis without skin manifestations under general anesthesia.

The clinical features of anaphylaxis are dependent on the type and administration route of the allergen [9]. The onset of anaphylaxis is fastest when the offending agent is given intravenously as a bolus, with life-threatening anaphylactic shock developing within minutes. In our first patient, respiratory symptoms developed within minutes after the administration of rocuronium. In the second patient, the onset of anaphylaxis following colloid infusion was slow, and it took more than 20 min after the commencement of dextran 40 infusion for the first symptoms to develop, although anaphylactic reactions to dextran have been reported to occur within a few minutes of starting an infusion [10]. There, thus, seems to be no full agreement on the onset time of anaphylaxis to dextran.

In previous reports, the incidences of cardiovascular symptoms, bronchospasm and cutaneous symptoms of anaphylaxis were reported to be 73.6–88.2, 37.3–44.2 and 69.6–74.6%, respectively [8, 11, 12]. Thus, the actual reported incidence of cutaneous manifestations seems to be approximately 70%, although widespread cutaneous manifestations are seen in the majority of cases of anaphylaxis during anesthesia [13]. In addition, according to a previous report, the most common initial features are pulselessness, difficulty in ventilation and desaturation [11]. These might indicate that cutaneous symptoms are not the initial presenting manifestations of anaphylaxis during general anesthesia in some cases. However, it is difficult to determine the initial symptoms of anaphylaxis during general anesthesia, because the patients are covered by surgical drapes. Even if anaphylaxis is detected by the appearance of respiratory or cardiovascular symptom, with skin manifestations being noticed thereafter, the skin manifestations might have already appeared before the appearance of respiratory or cardiovascular symptoms but may have remained unnoticed. In both our cases, however, the skin symptoms had not yet appeared when respiratory symptoms were detected. Hence, it is possible that the variabilities in cutaneous symptoms of anaphylaxis are still not fully elucidated.

Lieberman et al. reported that rapidly progressive anaphylaxis might be associated with a delay in skin manifestations [2]; however, they did not mention the interval between the initial symptoms and subsequent skin manifestations. In our cases, respiratory signs preceded the emergence of skin manifestations by approximately 10–15 min. Although skin manifestations are not the sole symptom of anaphylaxis, if the skin manifestations had developed earlier, the treatment of anaphylaxis with epinephrine administration could have been initiated earlier. Skin manifestations are reportedly present in 80–90% of anaphylaxis patients [1], and in less than 70% of cases of anaphylaxis during general anesthesia [2]. However, due to the delay in skin manifestations, the diagnosis of anaphylaxis during general anesthesia is often confusing. Further, although the absence of skin manifestations has been previously reported [14], delay in skin manifestations and its interval has not been the focus of many reports.

The reason for the delayed skin manifestations is thought to be the vasoconstriction induced by hypotension during rapidly progressive anaphylaxis [15]. In our cases, the initial sign was respiratory symptoms and the secondary sign was cardiovascular collapse. Skin manifestations appeared much later, before notable improvement in the hypotension. Therefore, neither the sequence of anaphylactic responses nor the actual mechanism of delayed skin manifestations during general anesthesia is adequately known.

In both our cases, serum tryptase concentrations were relatively higher during the event than 24 h after it. This observation is suggestive of anaphylaxis. Although an allergy test was not performed postoperatively in both cases, rocuronium and dextran, respectively, were considered to be the offending allergens due to the patients’ respective clinical courses.

A limitation of these case reports is that we did not perform allergy tests to conclusively determine the offending allergens. However, the diagnosis in both cases was made on the basis of identification of the three major clinical features of anaphylaxis and the interval between exposure to the suspected allergen and appearance of the initial symptoms, which, we believe, is reasonable. However, cases such as ours should be followed up by allergy testing.

## Conclusion

In both cases, there was a time lag between the appearance of respiratory and skin symptoms, which resulted in a delay in the diagnosis, and hence, treatment of anaphylaxis. Our experience highlights the fact that it is difficult to diagnose anaphylaxis under general anesthesia. We hope that our report will stimulate future large-scale research about the interval between the initial symptoms and subsequent skin manifestations of anaphylaxis.

## Abbreviations

ECG: electrocardiogram
BP: arterial blood pressure
HR: heart rate
SpO_2_: oxygen saturation
TCI: target controlled infusion
